# Imputation-Based Whole-Genome Sequence Association Study Rediscovered the Missing QTL for Lumbar Number in Sutai Pigs

**DOI:** 10.1038/s41598-017-00729-0

**Published:** 2017-04-04

**Authors:** Guorong Yan, Ruimin Qiao, Feng Zhang, Wenshui Xin, Shijun Xiao, Tao Huang, Zhiyan Zhang, Lusheng Huang

**Affiliations:** 1 State Key Laboratory for Pig Genetic Improvement and Production Technology, Jiangxi Agricultural University, 330045 Nanchang, P.R. China; 2 College of Animal Science and Veterinary Medicine, Henan Agricultural University, 450002 Zhengzhou, P.R. China

## Abstract

Resequencing a number of individuals of various breeds as reference population and imputing the whole-genome sequences of individuals that were genotyped with medium-density chips to perform an association study is a very efficient strategy. Previously, we performed a genome-wide association study (GWAS) of lumbar number using 60K SNPs from the porcine Illumina chips in 418 Sutai pigs and did not detect any significant signals. Therefore, we imputed the whole-genome sequences of 418 Sutai individuals from 403 deeply resequenced reference individuals and performed association tests. We identified a quantitative trait locus (QTL) for lumbar number in SSC1 with a *P value* of 9.01E-18 that was close to the potential causative gene of *NR6A1*. The result of conditioning on the top SNP association test indicated that only one QTL was responsible for this trait in SSC1. The linkage disequilibrium (LD) drop test result for the condition of the reported potential causative mutation (c.575T > C missense mutation of *NR6A1*) indicated that this mutation was probably not the underlying mutation that affected lumbar number in our study. As the first trial of imputed whole-genome sequence GWAS in swine, this approach can be also powerful to investigate complex traits in pig like in human and cattle.

## Introduction

Pigs were first domesticated from wild boars (*Sus scrofa*) approximately 10,000 years ago^1^. Thus, a large number of traits have changed dramatically, including more docile behavior, larger litter size and increased carcass length. The number of vertebrae associated with carcass length varies among breeds. Compared to the wild boar, European commercial pigs have 2–4 more vertebrae^2^. Because of its importance, this trait has received considerable attention. A quantitative trait locus (QTL) on chromosome 1 that affects the carcass in swine was identified in 1998 using a Meishan × White reciprocal backcross population^3^. In addition, a QTL significantly affecting vertebral number that is located extremely close to the QTL affecting carcass length on SSC1 was discovered in the Meishan × Gottingen cross population^4^. Furthermore, two additive quantitative trait loci (QTLs) on chromosome 1 and chromosome 7 were identified for the number of vertebrae using nine F2 families, including European breeds, Asian breeds, and miniature pigs^5^. Subsequently, to further investigate these two QTLs, fine mapping was carried out and the *NR6A1* gene was found to be a potential gene controlling the number of lumbar vertebrate; later, the c.575T > C missense mutation of this gene was suggested to be the potential mutation affecting the number of lumbar^6^. However, the QTL located on SSC1 was not detected in the Sutai population.

With the rapid development of SNP genotyping technology, genome-wide association studies (GWASs) have become a very effective and widely used approach to identify genetic variants associated with complex diseases or traits across the entire genome^7^. Using this strategy, several SNPs and QTLs and some quantitative trait genes (QTGs) were recently uncovered for economically important traits in pig breeds^8–11^. However, the power of GWASs is limited by the current density of SNP chips. The average density of the porcine SNP chips is much lower than the linkage disequilibrium (LD) block of most native breeds^12^, and as a result, several QTLs are missing from GWASs based on Illumina 60K porcine SNP chips. To improve the reliability and accuracy of GWASs, the use of high-density SNPs or even whole-genome sequence data to reperform the GWAS based on low-density SNPs is needed to identify missing QTLs. With the rapidly decreasing costs of next-generation sequence technology and the increasing accuracy of sequencing, numerous researchers have employed sequencing or resequencing to understand the demography, diversity and selection sweep of the investigated animals^13–15^. However, resequencing thousands of individuals and then determining associations for economically important traits is still an inefficient strategy. A more efficient approach is to impute the whole-genome sequence genotypes of individuals genotyped with medium-density chips using a previously sequenced reference population, and then determine associations between imputed genotypes and traits of interest using well-developed GWAS software. This approach is very popular for human disease studies, such as HapMap^16^ and the 1000 Genomes Project^17^, which provided standard reference panels. This approach has also worked very well in cattle, such as the 1000 bull genomes project (Run 2.0)^18, 19^. To the best of our knowledge, there are still no GWASs using whole-genome resequenced data in pigs.

Previously, we performed a GWAS using 60K porcine Illumina chips in Sutai pigs to detect the association loci for lumbar number. We expected to identify significant loci for this trait in Sutai pigs because this breed originated from Duroc and Erhualian pigs, which have similar paternal and maternal structures of an advanced intercross resource family^20^. Unexpectedly, no association signals were identified in Sutai pigs for lumbar number, which was different from the results of most published QTL mapping studies. Therefore, we hypothesized that the non-significant result may have arisen because of the low LD between causal mutation and nearby SNPs. To increase the detection power and decrease the cost of the GWAS, we first imputed the genotypes of 60K chips to the genotypes of whole-genome sequence variants in Sutai pigs using a reference panel containing 403 deep-sequenced individuals. Then, we used the imputed genotypes to reperform GWAS for the same phenotypes with the objective of determining whether there was a genetic variation in *NR6A1* associated with lumbar number in this breed. As noted above, the c.575T > C missense mutation of *NR6A1* was the strongest potential candidate for lumbar number. However, the causality of this SNP in Sutai pigs was unknown. In this study, we genotyped this mutation to estimate the imputation accuracy and its causality in Sutai pigs.

## Methods

### Ethics statement

All the experiments that involved animals were performed in accordance with the guidelines approved by the Ministry of Agriculture of China. Approval was obtained from the ethics committee of Jiangxi Agricultural University before this study.

### Animals of the target population

The target population of Sutai pig is a synthesized swine breed produced by crossing the Western Duroc and Chinese Erhualian breeds with continued selection for 19 generations. For the present study, we genotyped and phenotyped 526 individuals. The pigs were raised with the same fodder under uniform circumstances and slaughtered at 240 days of age in a commercial slaughterhouse. After the harvest, the carcasses were cut into halves and the numbers of lumbar vertebrae were counted and recorded. The lumbar number was either 5 or 6 in 436 pigs, including 206 gilts and 230 barrows, and the lumbar number was not available for 90 animals. More detailed information on the pigs’ environment and other phenotype data for these experimental animals were provided in our previous study^21^.

Genomic DNA samples were extracted from ear tissue using the standard phenol/chloroform method^22^, and the samples were diluted to a standardized concentration of 50 ng/µl after the quality was checked. A total of 526 samples were genotyped using Illumina PorcineSNP60 Beadchips, including 62,163 SNPs, on an iScan System (Illumina, San Diego, CA, USA)^23^. Quality control (QC) was conducted using PLINK (v1.90 beta) to detect and exclude unreliable genotypes^24^. SNPs with a missing rate of each marker (geno) >0.1 or with minor allele frequency (MAF) <0.05 were excluded. Individuals with a call rate <0.9 were also removed. To maintain consistency with the sequencing data, the primer sequences of each SNP were aligned to the reference porcine genome assembly Sus-scrofa 10.2 using BLAST to detect their positions and forward (reverse) strand information. SNPs without positions were excluded, and the genotypes of reversed SNP strands were flipped using PLINK software.

### Haplotype construction of the reference panel

In this study, a wide collection of 403 whole-genome sequence data from 10 different pig populations^15, 25–27^ was used as a reference and each breed contained 9 to 86 pigs. More details on the breeds, origins and sample size are listed in Table 1. The sequencing coverage of these individuals ranged from 5 to 25. The raw reads were cleaned based on a quality score threshold >15, which passed chastity filtering and would be then aligned to the reference porcine genome assembly Sus-scrofa 10.2 using BWA (Burrows-Wheeler Aligner)^28^. Variants were identified following the GATK (Genome Analysis Toolkit)^29^ best practice protocol. PCR duplications were first marked by Picard MarkDuplicates (http://broadinstitute.github.io/picard/), and local realignments were performed with GATK IndelRealigner. Individual GVCF files were produced using GATK Haplotypecaller. Variants were called and filtered with GATK Genotype GVCFs and VariantFiltration options. Structural variants were removed with VCFTOOLS^30^. With cleaned SNP data, the haplotypes of 403 individuals were constructed using Beagle (v4.1)^31^.

**Table 1:** The components of the reference panel.

Breeds	Sample Size	Coverage	Data Origin
Duroc	32	~25, 8	JXAU^*^, WAU^26^, Korea^27^
Erhualian	29	25	JXAU^15^^,*^
Large White	86	25, 8	JXAU^*^, WAU^26^, Korea^27^
Western Commercial	36	25, 8	JXAU^*^, WAU^26^, Korea^27^
CNH_Y	9	25, 8	JXAU^15^, WAU^26^
Wild Boar	34	25, 8, 5	JXAU^15^, WAU^26^, SCAU^25^
CNNorth	24	25	JXAU^15^^,*^
CNSouth	24	25	JXAU^15^^,*^
Tibetan	85	25, 5	JXAU^15^^,*^, SCAU^25^
CNElse	44	~25	JXAU^15^, WAU^26^, SCAU^25^

Breed and origin abbreviations:

CNH_Y: China Huai River and Yangtze River area pig; CNNorth: China North pigs; CNSouth: China South pigs; CNElse: China local pigs from other places.

JXAU: Jiangxi Agricultural University; WAU: Wageningen University; SCAU: Sichuan Agricultural University; Korea: Korea University.

*These part of data were sequenced by our laboratory and accessible under readers’ requirement.

### Imputation

Imputation from 60K SNPs to whole-genome sequences for Sutai pigs was conducted with Beagle (v4.1)^32^ using the default parameter settings, and the size of each sliding window was set to 7,000,000 bp. This software is based on a hidden Markov Chain Monte Carlo algorithm for imputation that first constructed local haplotypes using the MCMC algorithm and then resampled new estimated haplotypes for each individual using the HMM model.

Because of the very low density and common variants (MAF > 0.05) in 60K (Illumina, San Diego, CA, USA), imputation accuracy should be investigated in whole-genome sequence data. We used a 15-fold cross-validation strategy described in several previous studies^33–35^. Ninety individuals were selected randomly from the sequenced reference population as a target population for each fold (i.e. there would be some same individuals sampled in different target populations), and the genotypes in this target population were reduced to the variants that were included in the 60K genotyping array. The remaining individuals (313) were included in the reference panel. Two validation actions were taken to calculate the accuracy of imputation. One action was allelic correct rate (CR), which calculated as the number of alleles imputed correctly divided by total alleles at each locus, and the more detailed formula (see equation (1)) was as follows:

1 $$1-\frac{1}{2\times {\rm{m}}\times {\rm{{\rm N}}}}\sum _{i=1}^{m}\sum _{j=1}^{N}|{\rm{Obs}}({{\rm{n}}}_{ij})-{\rm{{\rm I}}}\mathrm{mp}({{\rm{n}}}_{ij})|$$

where m and N are the number of individuals and SNPs, respectively, and Obs (n_*ij*_) and Imp (n_*ij*_) are the observed and imputed numbers of allele “1” for individuals *i* at marker *j*, respectively. The other action was the correlation coefficient between true and imputed SNPs. To investigate the imputation accuracy impacted by MAF, we classified CR and correlation into 10 classes with regard to the MAF of imputed SNPs. The accuracy of imputation was the mean CR or correlation across 15 folds for each class.

### GWAS analysis

The associations between lumbar number and imputed genotypes were tested using GEMMA (v.0.93)^36^. This method implements a mixed model^37^ (see equation (2)) including covariates when we carried out conditional association test and LD drop association test, SNP effects, individual effects and residual error, which were calculated with the following formula:

2 $${\bf{y}}={\bf{W}}{\boldsymbol{a}}+{\bf{x}}{\boldsymbol{\beta }}+{\boldsymbol{u}}+{\boldsymbol{\varepsilon }};\quad {\bf{u}}\sim {{\rm{MVN}}}_{n}({\rm{0}},\lambda {\tau }^{-1}{\bf{K}}),\quad {\boldsymbol{\varepsilon }}\sim {{\rm{MVN}}}_{n}(0,{\tau }^{-1}{{\bf{I}}}_{n})$$

where **y** is the vector of phenotypes; **W** is a matrix of covariates, including a column of 1s; ***α*** is a vector of the corresponding coefficients, including the intercept; **x** is a vector of genotypes; ***β*** is the effect of markers; **u** is a vector random effect following the multivariate normal distribution (see equation (2)), in which *τ* ^−1^ is the variance of the residual errors, λ is the ratio between *τ* ^−1^ and ε, and K is a kinship matrix that is estimated from whole-genome sequence variants; ε is a vector of errors following the multivariate normal distribution (see equation (2)) and **I**_*n*_ is an identity matrix. Using naïve Bonferroni corrections of 0.05 divided by the number of examined SNPs would lead to an overly conservative threshold because these SNPs were highly correlated with each other. Pe’er *et al*. and Johnson *et al*. suggested that 5E-08 could serve as a genome-wide significant threshold in human GWASs based on haplotype blocks of an African population structure^38, 39^. Based on the assumption that an equal number of independent haplotype segments between pigs and humans are held, we used the same genome-wide threshold in our study. The model for the GWAS of Sutai pigs with 60K genotypes was the same as that used for whole-sequence association tests and the kinship matrix was estimated either from 60K SNPs (original SNP-data) or whole-genome sequence variants. To make the results comparable, the values of the 60K marker from the results of the whole-sequence association study were extracted for comparison.

### LD analysis

To detect the linkage disequilibrium (LD) of SNPs near the most significant SNPs in the GWAS results, the 3 Mb region near the top SNPs in the whole-sequence association results was used to conduct LD analysis by extracting genotypes from the 60K data set using Haploview (v.4.2) software^40^. Haplotype blocks were then estimated with a confidence intervals algorithm in Haploview.

### Genotyping of c.575T > C locus

Variation of the c.575T > C (rs326780270) of *NR6A1* in Sutai pigs was detected following the methods of Yang *et al*.^41^. Briefly, a 360 bp segment was amplified and cut into two pieces of 183 and 177 bp for allele C at the position of 299,084,752 bp on SSC1. Genotypes of this locus were then identified through agarose gel electrophoresis.

### Conditional association test

To elucidate whether there are additional QTLs for lumbar number on SSC1, we performed a conditional test by including the genotypes of the top SNPs as a covariance to the mixed model and retested the association between SNPs and phenotypes. If no additional signal was detected, then there was only one QTL that affected lumbar number. Otherwise, there were multiple QTLs that cooperated to control lumbar number.

### LD drop association test

To determine whether *NR6A1* c.575T > C was the mutation that determined lumbar number in Sutai pigs, we performed an LD drop test by including the genotypes of *NR6A1* c.575T > C in the mixed model framework to determine how rapidly the association with the signal decreased.

## Results

### SNP characteristics after QC in the target panel

After QC, 11,338 variants were excluded for the lack of chromosome position information, 42 pigs were removed due to a low genotype call rate, 3,229 variants were removed due to a low call rate and 9,804 variants were excluded for low minor allele threshold(s). Finally, a total of 37,792 SNPs and 484 pigs were introduced to perform further analyses.

### Summary of imputation

**Figure 1:**
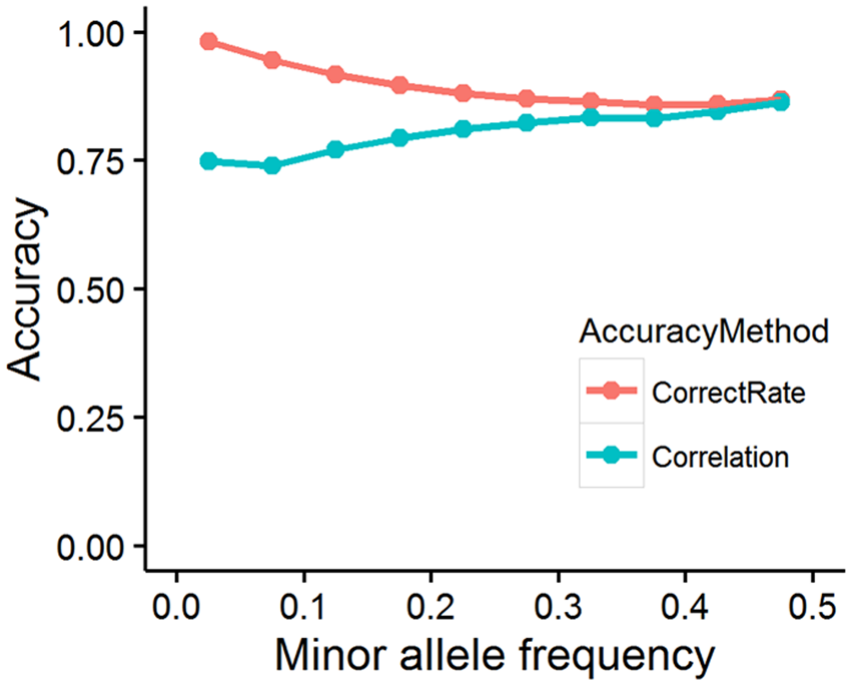
Evaluation of imputation accuracy MAF. The x-axis is the MAF range from 0 to 0.5, and the y-axis is imputation accuracy denoted by the correct rate (CR) and correlation. The pink line shows the CR, which was calculated as the number of alleles imputed correctly divided by the total alleles at each locus across MAF. The blue line shows the correlation between true and imputed genotypes at each locus across MAF.

Imputation was produced using Beagle software. The summarization of imputation results is presented in Table 2. After imputation, we obtained 87,552,595 SNPs for 484 individuals, and 20,985,704 SNPs were kept after filtering with MAF > 0.01. SSC1 was selected for 15-fold cross-validation to calculate the imputation accuracy tested by CR and correlation related to MAF. The correct rate decreased when MAF increased. In contrast, the correlation increased along with the increase of MAF (Fig. 1). The average CR was 0.90 with maximum and minimum values varying from 0.98 to 0.86 across MAF. The average correlation was 0.80 with maximum and minimum values ranging from 0.86 to 0.74.

**Table 2:** The distribution of SNPs in different chromosomes.

Chr	Before QC (SNP/NIND)	After QC (SNP/NIND)
Chr 1	9,369,975/484	1,930,649/418
Chr 2	5,734,943/484	1,430,107/418
Chr 3	4,910,467/484	1,242,704/418
Chr 4	4,774,170/484	1,139,870/418
Chr 5	3,816,805/484	961,177/418
Chr 6	5,216,961/484	1,264,513/418
Chr 7	4,663,028/484	1,153,610/418
Chr 8	5,035,221/484	1,185,766/418
Chr 9	5,392,245/484	1,344,280/418
Chr 10	3,405,060/484	1,028,000/418
Chr 11	3,347,457/484	853,465/418
Chr 12	2,406,736/484	660,770/418
Chr 13	7,186,391/484	1,442,485/418
Chr 14	5,407,899/484	1,282,555/418
Chr 15	4,998,888/484	1,105,999/418
Chr 16	3,266,711/484	815,314/418
Chr 17	2,608,589/484	686,832/418
Chr 18	2,373,396/484	602,229/418
Chr 19	3,637,653/484	855,379/418
Whole genome	87,552,595/484	20,985,704/418

**Chr**: chromosome number; **QC**: quality control. the QC condition was MAF > 0.01 and 66 individuals were removed for the case of without phenotypes.

### Summary of GWAS

**Figure 2:**
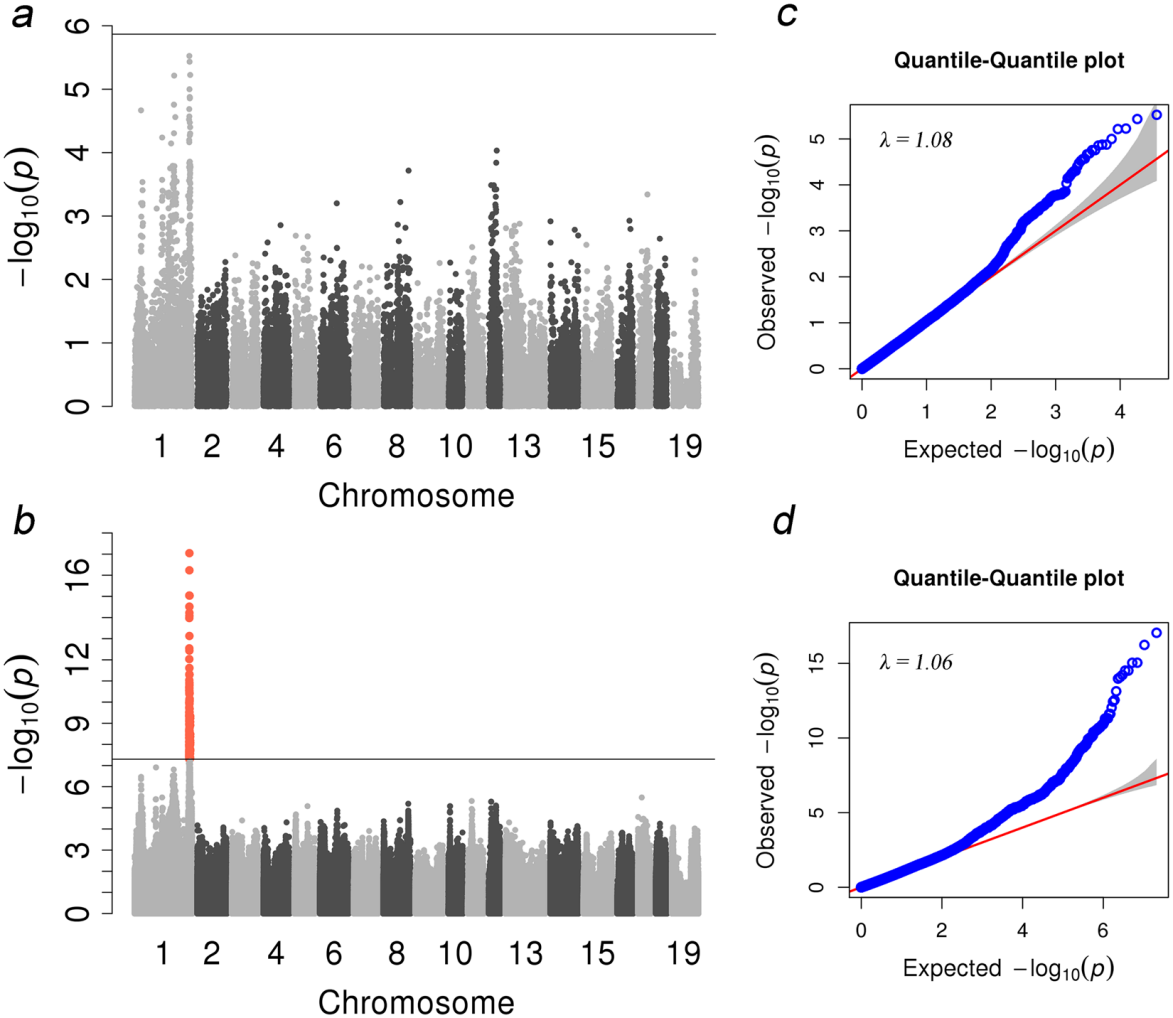
GWAS results for lumbar number trait. (**a**,**b**) Manhattan plots for lumbar number with the data before imputation (**a**) and after imputation (**b**). (**c**,**d**) c and d are the quantile-quantile plots. In the Manhattan plots, the y-axis and x-axis represent the negative log_10_ *P value* of the SNPs and the genomic positions separated by chromosomes, respectively. In Manhattan plot a, black solid lines indicate the 5% genome-wide Bonferroni-corrected threshold. In Manhattan plot b, the tomato puree points represent SNPs that exceeded the chromosome-wide significance threshold (−log10(5E-08)), and the black solid lines indicate the significance threshold. In quantile-quantile plots c and d, the y-axis and x-axis represent the expected and observed negative log_10_ *P value*s, respectively.

We conducted a GWAS on the Sutai population in two scenarios, i.e., the target panel data before and after imputation. In the scenario for before imputation, as noted above, no significant loci were detected in Sutai pigs using 60K chips (Fig. 2a, which contains the 60K original data), and *P values* positioned on the 60K original data were extracted from sequencing GWAS (which included the 60K imputed data). To further compare array based result to sequences based result underlying the same kinship matrix, we extracted *P values* positioned on the 60K chips from result of sequencing GWAS. The Manhattan plots of the 60K imputed data results are shown as Supplementary Fig. S1. Both results confirmed that no significant QTLs were located on SSC1 when only 60K SNPs were used. The association *P values* of the top SNP in the 60K imputed data and the 60K original data were 1.27E-06 and 2.99E-06, and the position of the top SNP in both results was 298972575 (rs81352477) in chromosome 1. In the scenario with the sequence data, 105 genome-wide significant SNPs were uncovered (Table 3, Fig. 2b) on SSC1 within a 4.6 Mb region (298,912,325 bp-303,530,285 bp). Furthermore, the proposed causal gene, *NR6A1*, for lumbar number^6^ was located in this region. However, the *P value* of the proposed causal mutation c.575T > C was only 2.26E-06 at the position of 299,084,752 bp (imputation accuracy, r^2^ = 0.95), which indicated much lower significance than the top SNP (*P value* = 9.01E-18) at a position of 299,627,873 bp.

**Table 3:** Description of the most significant 20 SNPs associated with lumbar number by GWAS.

Chr	rs	ps	n_miss	beta	se	l_remle	l_mle	p_wald
Chr 1	rs334252332	299,627,873	0	3.02E-01	3.35E-02	1.00E-05	1.00E-05	9.01E-18
Chr 1	rs331286845	299,560,236	0	3.12E-01	3.56E-02	6.58E-04	1.00E-05	5.84E-17
Chr 1	rs344688372	299,031,889	0	2.92E-01	3.48E-02	6.41E-02	1.46E-02	9.09E-16
Chr 1	rs333213419	300,706,429	0	3.01E-01	3.59E-02	9.87E-03	1.00E-05	9.22E-16
Chr 1	rs336248841	299,463,071	0	2.76E-01	3.36E-02	1.00E-05	1.00E-05	3.06E-15
Chr 1	rsxxxxxxxx1	299,590,806	0	3.01E-01	3.66E-02	1.00E-05	1.00E-05	3.06E-15
Chr 1	rs341631790	299,554,614	0	3.03E-01	3.74E-02	4.42E-02	1.00E-05	6.01E-15
Chr 1	rs320822074	299,569,286	0	2.98E-01	3.69E-02	1.91E-02	1.00E-05	8.34E-15
Chr 1	rs326834750	299,031,654	0	2.77E-01	3.45E-02	4.60E-02	1.00E-05	1.06E-14
Chr 1	rs334124688	299,663,720	0	2.91E-01	3.74E-02	7.20E-02	3.82E-04	7.46E-14
Chr 1	rs327909125	299,031,891	0	2.79E-01	3.70E-02	2.02E-01	1.69E-01	2.86E-13
Chr 1	rs329239802	299,464,519	0	2.72E-01	3.62E-02	6.56E-02	1.00E-05	3.67E-13
Chr 1	rs320616940	298,988,212	0	2.65E-01	3.58E-02	1.18E-01	1.00E-05	9.25E-13
Chr 1	rs319146997	299,462,559	0	2.62E-01	3.62E-02	7.32E-02	1.18E-02	2.45E-12
Chr 1	rs331600883	299,462,537	0	2.62E-01	3.62E-02	7.32E-02	1.18E-02	2.45E-12
Chr 1	rs334129807	299,554,649	0	2.83E-01	3.96E-02	1.61E-01	1.29E-01	4.86E-12
Chr 1	rs324516984	299,741,083	0	2.85E-01	3.99E-02	1.56E-01	1.18E-01	4.89E-12
Chr 1	rsxxxxxxxx2	299,561,306	0	2.86E-01	4.01E-02	1.77E-01	1.45E-01	5.05E-12
Chr 1	rs323786500	299,562,197	0	2.86E-01	4.01E-02	1.77E-01	1.45E-01	5.05E-12
Chr 1	rs320840172	298,943,126	0	2.47E-01	3.51E-02	8.59E-02	1.00E-05	9.23e-12

**Chr**: chromosome number; **rs**: SNP IDs and two SNPs that do not possess rs ID were named after rsxxxxxxxx1 and rsxxxxxxxx2, respectively, by the author; **ps**: base pair positions on the chromosome; **n_miss**: number of missing values of the SNP; **beta**: beta estimates; **se**: standard errors for beta; **l_remle**: remle estimates for lambda; **l_mle**: mle estimates for lambda; **p_wald**: *P value* from the Wald test.

### LD results

**Figure 3:**
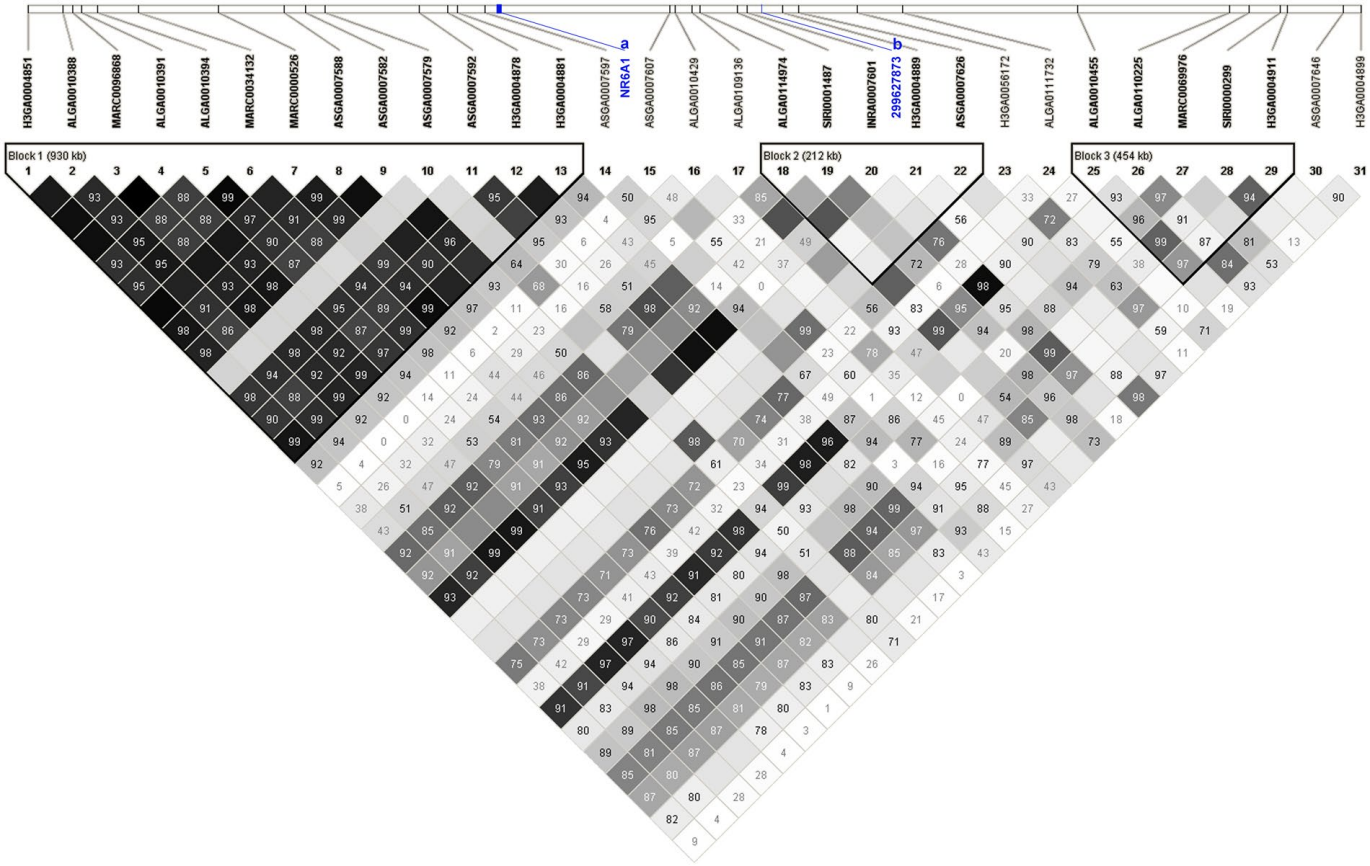
Haplotype block of a significant region (3 Mb) of SSC1 in Sutai pigs. The *NR6A1* gene (**a**) did not fall into any block in this region, and the most significant position (**b**) was located at 299,627,873 bp in the 454 kb block 3.

By carrying out GWAS with imputation data, we identified the most significant SNP at a position of 299,627,873 bp as well as a total of 31 markers that were extracted from the significant region (3 Mb) in the 60K data that were used to conduct LD analysis. The LD block was shown as follows (Fig. 3). Three blocks were detected on this region using a confidence interval algorithm. The most significant was the smallest block of approximately 212 kb, and the r^2^ among each SNP in this region was very low. The *NR6A1* gene was not present in any block in this region.

### Results of genotyping c.575T > C

Among the 526 samples, a total of 382 pigs were genotyped on the c.575T > C locus. Subsequently, we obtained 187 CC genotypes, 166 CT genotypes and 29 TT genotypes (see Supplementary Table S1). To further confirm imputation accuracy, we compared imputed genotypes to real genotyped genotypes on this locus and found that only 12 of 382 individuals had different genotypes. In other words, a very high allelic imputation accuracy (98.43%) was obtained at this locus.

### Results of the conditional association test and LD drop association test

**Figure 4:**
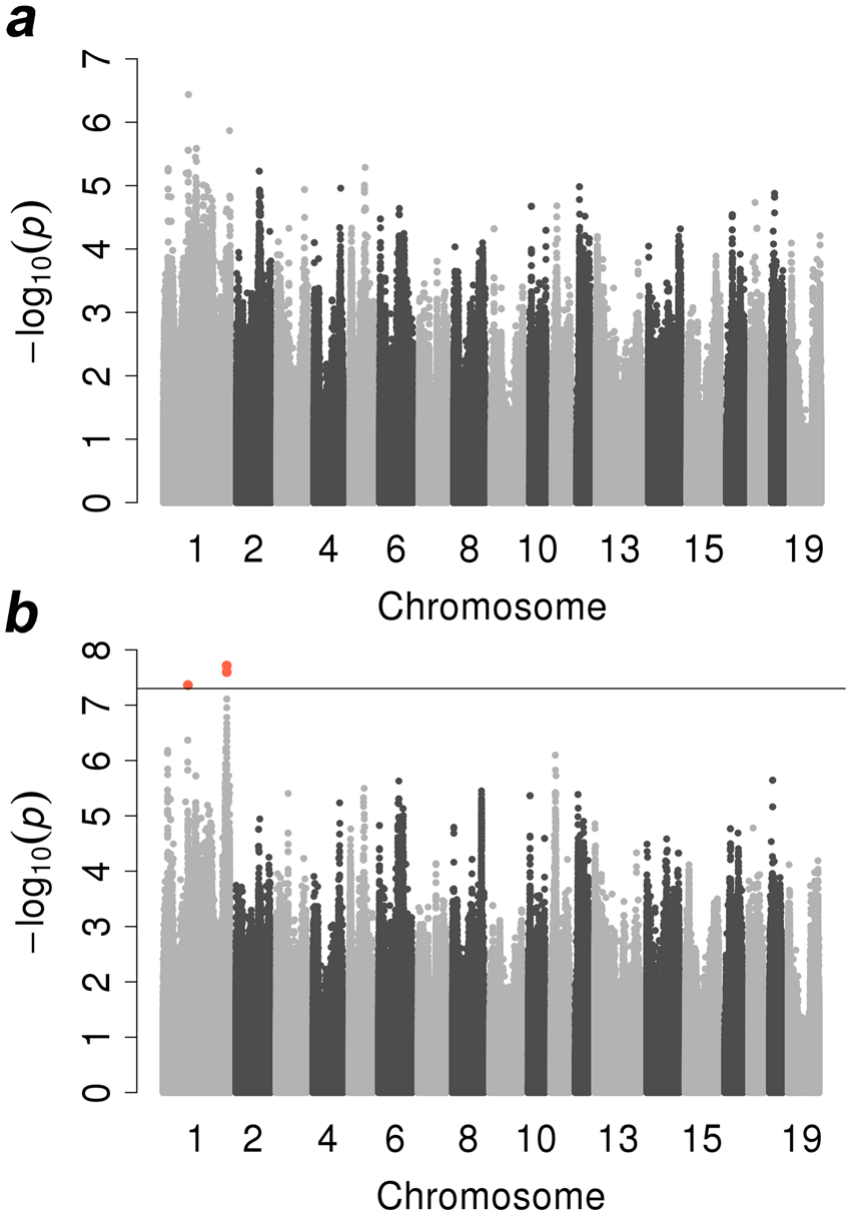
GWAS results for lumbar number in two scenarios: conditional test and LD drop test. (**a**,**b**) Manhattan plots for lumbar number in the conditional association test and LD drop association test, respectively. In the Manhattan plots, the y-axis and x-axis represent the negative log_10_ *P values* of the SNPs and the genomic positions separated by chromosomes, respectively. In Manhattan plots a and b, the black solid lines indicate the chromosome-wide significance threshold (−log10(5E-08)), and in (**b**), the tomato puree points represent SNPs that exceeded the chromosome-wide significance threshold.

After GWAS was performed by including the most significant SNP from imputed GWAS results in a mixed model as a covariate, no additional genome-wide significant loci were detected on this chromosome, which indicated that only one major QTL affected lumbar number (Fig. 4a).

After fitting genotypes of *NR6A1* c.575T > C into the mixed model for the LD dropping test, we still identified a genome-wide significant locus near the top SNP at a position of 299,432,549 bp with a *P value* of 1.93E-08. This result probably indicated that locus *NR6A1* c.575T > C was not the causative mutation in Sutai pigs for lumbar number (Fig. 4b).

## Discussion

Imputation-based association studies have achieved great success in humans^42–45^ and some livestock, such as cattle^46^. Both have resequenced more than 1000 individuals of multiple populations as reference panels, and the unrelated targets were genotyped using middle- (high-) density SNP chips. Whole-genome sequences of the target panel were imputed based on shared haplotype blocks between reference and target individuals and then were used to test associations of complex disease (traits) or to predict the genetic potential of economically important traits. Imputation accuracy ranged from 0.90 to 0.95 in cattle from the genotypes of an Illumina BovineHD genotyping array to whole-genome sequence data^35^. High correlations (0.64) were observed in humans with MAF = 0.1% when imputing an Illumina 1M SNP array to whole-genome sequences using a reference panel of 64,976 haplotypes^45^. In our study, a total of 403 individuals were included on the reference panel, including 32 Duroc and 29 Erhualian pigs, which are ancestors of the Sutai population. CR decreased and correlation increased along with an increase in MAF. CRs are highly sensitive to allelic frequencies and are not appropriate for comparing SNPs with different values of MAF^47^. Correlation is a more popular approach that is used to evaluate imputation accuracy. The correlation values ranged from 0.74 to 0.86 with an average of 0.80, which was lower than the results for cattle and human studies. Both were imputed from high-density chips (600K in cattle and 1M in human) to sequenced data, and the reference panels were very large. In pigs, the vast majority of studies were based on genotypes from the 60K porcine Illumina BeadChip because a high-density panel (600,000 SNPs) that provides high-quality imputed genotypes in pig populations is currently impractical. Therefore, increasing the number of sequenced populations and individuals in the reference panel to improve imputation accuracy is necessary. Our GWAS results demonstrated that this was a powerful method to identify QTLs in agricultural animals, and this method will help researchers find new loci or rediscover QTLs associated with complex traits.

Since the first application of GWAS research on age-related macular degeneration was performed successfully in 2005 by Klein *et al*.^48^, GWAS has become an effective method for identifying genetic variations associated with economically important traits in agricultural animals. A recent GWAS study showed that the QTL for the number of vertebrae on chromosomes 1 and 7 independently influenced the numbers of thoracic and lumbar vertebrae^49^. Potentially significant signals could be missed in a GWAS analysis if low-density SNPs were applied to a population that held a low LD characteristic, such as the results of our GWAS when only 60K SNPs were used before imputation and a highly significant QTL was uncovered for lumbar number after imputation. The LD between top SNPs in the 60K original association results (rs81352477) and top SNPs (rs334252332) in the sequence association result was 0.75, indicating a medium correlation. The increased detection power was probably due to causal mutations being in the data by imputation. This result was further confirmed by displaying the LD profiles of markers near the top loci. The top SNP was located in the smallest haplotype block, and the r^2^ values among these SNPs in this region were very low, which hampered the discovery of association signals. Furthermore, no haplotype block was found near the *NR6A1* gene, which implicated the low LD station in that region in the Sutai population. The Sutai breed was intercrossed from Erhualian female and Duroc male for approximately 19 generations. Thus, the Sutai genome is a mosaic mixture of these two breeds. As a result, the LD block is smaller than the LD block in either of the two founder breeds. In this study, we identified 105 significant SNPs located on chromosome 1 across a region of 4.6 Mb (298,912,325 bp–303,530,285 bp) associated with lumbar number, and the highest signal was located on 299,627,873 bp of chromosome 1. This region contains the *NR6A1* gene, which was reported to be associated with lumbar number^6^. The results also showed that using whole-genome resequencing data to perform genotype imputation can be an effective method to identify the QTLs that were missed in low-density SNP GWAS analysis. The imputation method can also narrow the QTL region or improve the power when GWAS analysis is performed. To determine whether population stratification was corrected in this study, we exploited quantile-quantile plots (Fig. 2c and d) from the GWAS with 60K SNP data and imputed the sequenced data. The two quantile-quantile plots with lambda values of 1.08 and 1.06 showed that the population stratification effect was adjusted very well, and the detected signal was most likely reliable.

Although we identified the same QTL as that identified in a previous study^4^, the reported potential causative mutation at position of 299,084,752 bp (c.575T > C)^6^ showed only a weak association with lumbar number in our study (*P value* = 2.26E-06). The possible reason for this result is that the QTN in the position of 299,084,752 bp may not be the causative mutation in the Sutai population. To confirm that *NR6A1* c.575T > C was the causative mutation in our population, we performed an LD drop test by fitting genotypes of this locus into a mixed model. Normally, all significant signals nearby would disappear after correcting for causative mutation. The minimum *P value* increased from 9.01E-18 to 1.93E-08, which still indicated genome-wide significance. This result indicated that *NR6A1* c.575T > C was not the causative mutation in our study. The results also indicated that we should recognize that the accuracy of imputation also affects the GWAS result. Several imputation studies in different species have shown that as the minor allele frequency in the target panel decreased, the imputation error rate increased^34, 50^. As shown in previous studies, the fundamental aspect of imputation is the identical DNA segments in the target and reference panels, and increasing the number of parents or male parents in a reference panel can increase the imputation accuracy^51–53^. In other words, if we can increase the number of individuals in the target panel and reference panel, the imputation accuracy will be increased. Mixing different breeds in a reference panel would thus improve imputation accuracy^54^. In this study, we mixed different pig breeds in the reference panel and executed strict quality control, such as MAF and call rate, in the target and reference panels. We achieved a high CR with an average of 90% and real genotypes of c.575T > C, which confirmed the high imputation accuracy (98.43%). Therefore, this factor may not be very critical in this study, but we also should pay more attention to exploring the factors that affect the imputation results in the future. In addition, a reassociation study using real genotypes at c.575T > C achieved a *P value* of only 3.89E-07, which further indicates that it was not associated with lumbar number in the Sutai population. To determine whether there are several causative mutations responsible for lumbar number, we performed a conditional test by adjusting the top SNP on SSC1 and conducted GWAS again. Additional significant signals would stand out if the multiple causative mutation hypothesis was true. In our analysis, there were no other QTLs associated with lumbar number, which means there is only one QTL that controls lumbar number on SSC1, whereas the causative mutation is not the same as that previously reported. Further functional studies, such as gene expression and site-specific editing technology, are necessary to confirm the possibility of causality for the top SNP in the Sutai population.

In this study, we rediscovered the missing QTL for lumbar number in Sutai pigs using GWAS based on a whole-genome imputation strategy. This QTL includes the same potential causative gene, *NR6A1*, that was previously reported, while the top SNP differed from the previously reported potential causative mutation. This study illustrates the importance and effectiveness of uncovering the traits in agricultural animals using a whole-genome imputation approach and provides a solution that combines second-generation sequence data with GWAS. Our results also show that this approach can be a powerful strategy to analyze economically important complex traits in livestock. Along with developing good imputation software, exploiting more public database systems will contribute to genotype imputation in the future.

## Electronic supplementary material

Supplementary info
