# Supplementary material for: Imputation-Based Whole-Genome Sequence Association Study Rediscovered the Missing QTL for Lumbar Number in Sutai Pigs
Source: Sci Rep. 2017 Apr 4;7:615. doi: 10.1038/s41598-017-00729-0 (PMC5429657; doi:10.1038/s41598-017-00729-0)
Supplement: Supplementary file 1 — Supplementary info [file 41598_2017_729_MOESM1_ESM.pdf]

SUPPLEMENTARY INFORMATION

Imputation-Based Whole-Genome Sequence Association Study Rediscovered the Missing QTL for Lumbar Number in Sutai Pigs

Guorong Yan[1,†], Ruimin Qiao[2,†], Feng Zhang[1], Wenshui Xin[1], Shijun Xiao[1], Tao Huang[1], Zhiyan Zhang[1,*], Lusheng Huang[1]

[1]State Key Laboratory for Pig Genetic Improvement and Production Technology, Jiangxi Agricultural University, 330045, Nanchang, P.R. China

[2]College of Animal Science and Veterinary Medicine, Henan Agricultural University, 450002, Zhengzhou, P.R. China

[†] these authors contributed equally to this work

[*] Corresponding author bioducklily@hotmail.com (Z. Y. Zhang)

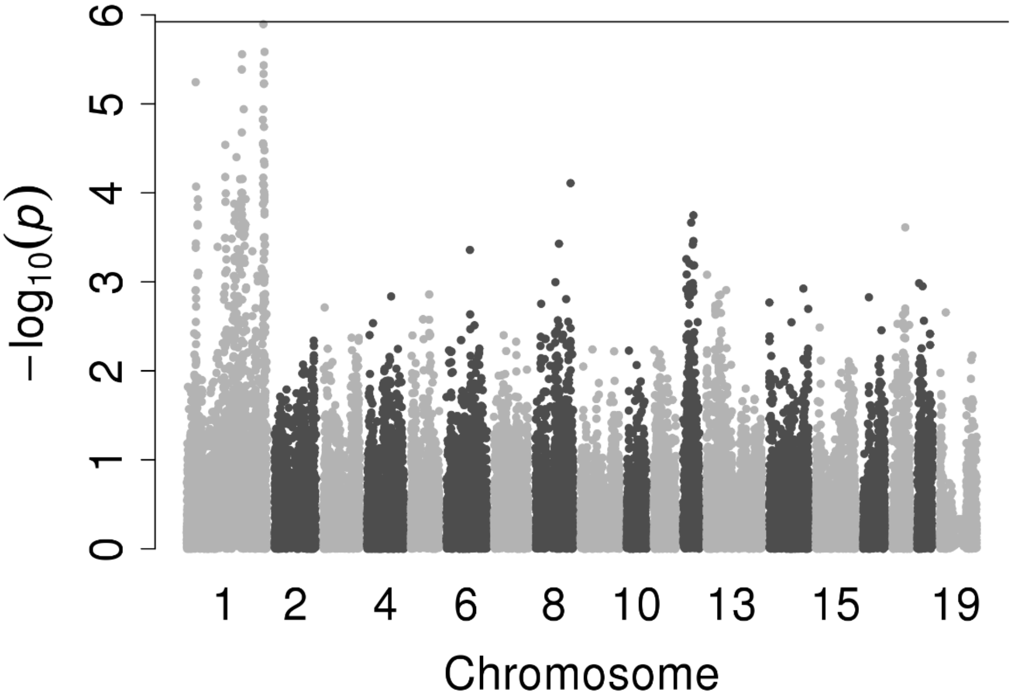

Supplementary Figure S1

GWAS result of 60k chip SNPs extracted from sequencing GWAS result. In the Manhattan plots, the y-axis and x-axis represent the negative log10 *P value* of the SNPs and the genomic positions separated by chromosomes, respectively. The gray solid lines indicate the 5% genome-wide Bonferroni-corrected threshold.

Supplementary Table S1.

Comparison of sequenced and imputed genotypes in the locus of c.575T>C.

Imputed \ Sequenced	CC	CT	TT
**CC**	182	5	0
**CT**	5	159	0
**TT**	0	2	29
